# LightGBM hybrid model based DEM correction for forested areas

**DOI:** 10.1371/journal.pone.0309025

**Published:** 2024-10-07

**Authors:** Qinghua Li, Dong Wang, Fengying Liu, Jiachen Yu, Zheng Jia

**Affiliations:** College of Geodesy and Geomatics, Shandong University of Science and Technology, Qingdao, Shandong, China; USACE ERDC: US Army Engineer Research and Development Center, UNITED STATES OF AMERICA

## Abstract

The accuracy of digital elevation models (DEMs) in forested areas plays a crucial role in canopy height monitoring and ecological sensitivity analysis. Despite extensive research on DEMs in recent years, significant errors still exist in forested areas due to factors such as canopy occlusion, terrain complexity, and limited penetration, posing challenges for subsequent analyses based on DEMs. Therefore, a CNN-LightGBM hybrid model is proposed in this paper, with four different types of forests (tropical rainforest, coniferous forest, mixed coniferous and broad-leaved forest, and broad-leaved forest) selected as study sites to validate the performance of the hybrid model in correcting COP30DEM in different forest area DEMs. In the hybrid model of this paper, the choice was made to use the Densenet architecture of CNN models with LightGBM as the primary model. This choice is based on LightGBM’s leaf-growth strategy and histogram linking methods, which are effective in reducing the data’s memory footprint and utilising more of the data without sacrificing speed. The study uses elevation values from ICESat-2 as ground truth, covering several parameters including COP30DEM, canopy height, forest coverage, slope, terrain roughness and relief amplitude. To validate the superiority of the CNN-LightGBM hybrid model in DEMs correction compared to other models, a test of LightGBM model, CNN-SVR model, and SVR model is conducted within the same sample space. To prevent issues such as overfitting or underfitting during model training, although common meta-heuristic optimisation algorithms can alleviate these problems to a certain extent, they still have some shortcomings. To overcome these shortcomings, this paper cites an improved SSA search algorithm that incorporates the ingestion strategy of the FA algorithm to increase the diversity of solutions and global search capability, the Firefly Algorithm-based Sparrow Search Optimization Algorithm (FA-SSA algorithm) is introduced. By comparing multiple models and validating the data with an airborne LiDAR reference dataset, the results show that the R^2^ (R-Square) of the CNN-LightGBM model improves by more than 0.05 compared to the other models, and performs better in the experiments. The FA-SSA-CNN-LightGBM model has the highest accuracy, with an RMSE of 1.09 meters, and a reduction of more than 30% of the RMSE when compared to the LightGBM and other hybrid models. Compared to other forested area DEMs (such as FABDEM and GEDI), its accuracy is improved by more than 50%, and the performance is significantly better than other commonly used DEMs in forested areas, indicating the feasibility of this method in correcting elevation errors in forested area DEMs and its significant importance in advancing global topographic mapping.

## Introduction

The digital elevation model in forested areas plays a crucial role across various domains. Its applications encompass terrain analysis, including slope, elevation variation, and topographic relief, providing key information for studying the influence of terrain on hydrological processes and ecosystems. Light detection and ranging (LiDAR) data are collected from satellites or other platforms by sensors that detect the reflection of pulsed laser beams. These reflections are recorded as "point clouds" that represent the 3D location of buildings, vegetation and the ground. A Digital Elevation Model (DEM) is one of the products derived from LiDAR data. By stripping surface features and uniformly sampling ground elevation, a high-resolution DEM can be derived from the point cloud data to produce a bare ground model. LiDAR DEM, being considered the most ideal choice, is associated with high costs for laser surveying, and the fact that many governments do not publicly provide LiDAR DEM makes it difficult to obtain such DEM on a global scale. Therefore, acquiring global forest DEMs relies on satellite remote sensing images, such as AW3D30 DEM, SRTM, ASTER GDEM, COP30 DEM, and NASA DEM based on SRTM correction. Taking NASA DEM as an example, the root mean square error (RMSE) in the Estonia region is 6.39 meters [1]. In the Fenhe River Basin in China, the RMSE of ASTER GDEM is 5.89 meters [2]. For vegetated areas, the accuracy of SRTM is approximately 6 meters [3], while in the forested areas in the northeast of Mindoro Island in the Philippines, the RMSE of AW3D DEM reaches 6.67 meters [4]. It is also found that COP30DEM’s accuracy in terrain height is superior to that of AW3D30, NASA DEM, and SRTM [5]. Guth et al. validated the aforementioned global DEMs in forested areas using LiDAR point clouds and ICESat-2, obtaining consistent results with the above studies [6].

Currently, scholars are deeply researching how to evaluate and correct DEMs in forested areas with LiDAR technology. Their work covers various aspects. For instance, SU et al. assessed and corrected SRTM elevation errors using LiDAR data [7]. However, due to the limited coverage of airborne data, the global application of this method is restricted. To address this problem, some scholars adopted a combined approach using different data sources. They combined SRTM DEM with ICESat ground points and MODIS satellite tree cover to generate a global bare-earth DEM [8]. LI et al. corrected a forest DEM by neural network, taking into account spatial autocorrelation [9]. Fayad et al. estimated canopy height in French Guiana using machine learning techniques based on ICESat/GLAS data [10]. With the release of ICESat-2 data, researchers have begun exploring its application in DEM correction. For example, Zhu et al. used ICESat-2 data for terrain slope inversion [11]. Li et al. generated topographic data for Massachusetts from ICESat-2 data and combined it with SRTM DEM interpolation [12]. They also corrected ALOS PALSAR DEM reference data based on ICESat-2 [13]. Additionally, Liu et al. evaluated the performance of GEDI and ICESat-2 laser altimeter data in terrain and canopy height inversion [14]. Overall, the research findings of numerous scholars indicate that spaceborne LiDAR data have extensive applicability in globally correcting forest DEMs, addressing limitations such as limited coverage of airborne LiDAR data and insufficient accuracy of optical satellite data.

Although some progress has been made in the correction and inversion of digital elevation models, current research still faces challenges and limitations. Firstly, it is observed that the original data of the calibrated DEMs often exhibit lower precision, thereby constraining the potential for enhancing the final model accuracy, in view of which the COP30DEM data, which is a more accurate version of the original DEM data, was chosen for this paper. Secondly, given the diverse range of forest covers globally, it is imperative to ensure that our research methodology is applicable across various regions and different types of forest environments. Such broad applicability is crucial for practical applications and global-scale research. Therefore, designing research methods that are broadly applicable and ensuring that they can be effectively applied in a variety of forest environments enhances their value for practical application and global-scale research. Thirdly, the current correction process typically uses a single model or traditional ensemble learning models without combining ensemble learning with deep learning hybrid models for forest DEM correction. Hybrid models have achieved significant success in other fields, so applying them to forest DEM correction may yield better results and higher accuracy. Therefore, the innovative combination of ensemble learning and deep learning hybrid models overcomes the limitations of traditional approaches in model selection. Finally, despite its low resolution in the perpendicular-to-orbit direction, the data coverage of the on-board ICESat-2 is global and far exceeds the coverage of currently available airborne LiDAR data. Therefore, it has better global applicability as a true value for DEM correction. Therefore, based on data from COP30DEM and ICESat-2, attempting to use hybrid learning models to obtain forest digital elevation models. In this method, we use ICESat-2 provided ground height as the true value, regressively correcting COP30DEM elevation data to obtain a more accurate forest digital elevation model. Our research focuses on comparing the results of various models to evaluate their performance differences in vegetation correction and hopes to provide more effective tools and methods for accurately describing and understanding forest terrain in the future.

## Test sites and data preparations

### Test sites

**Fig 1:**
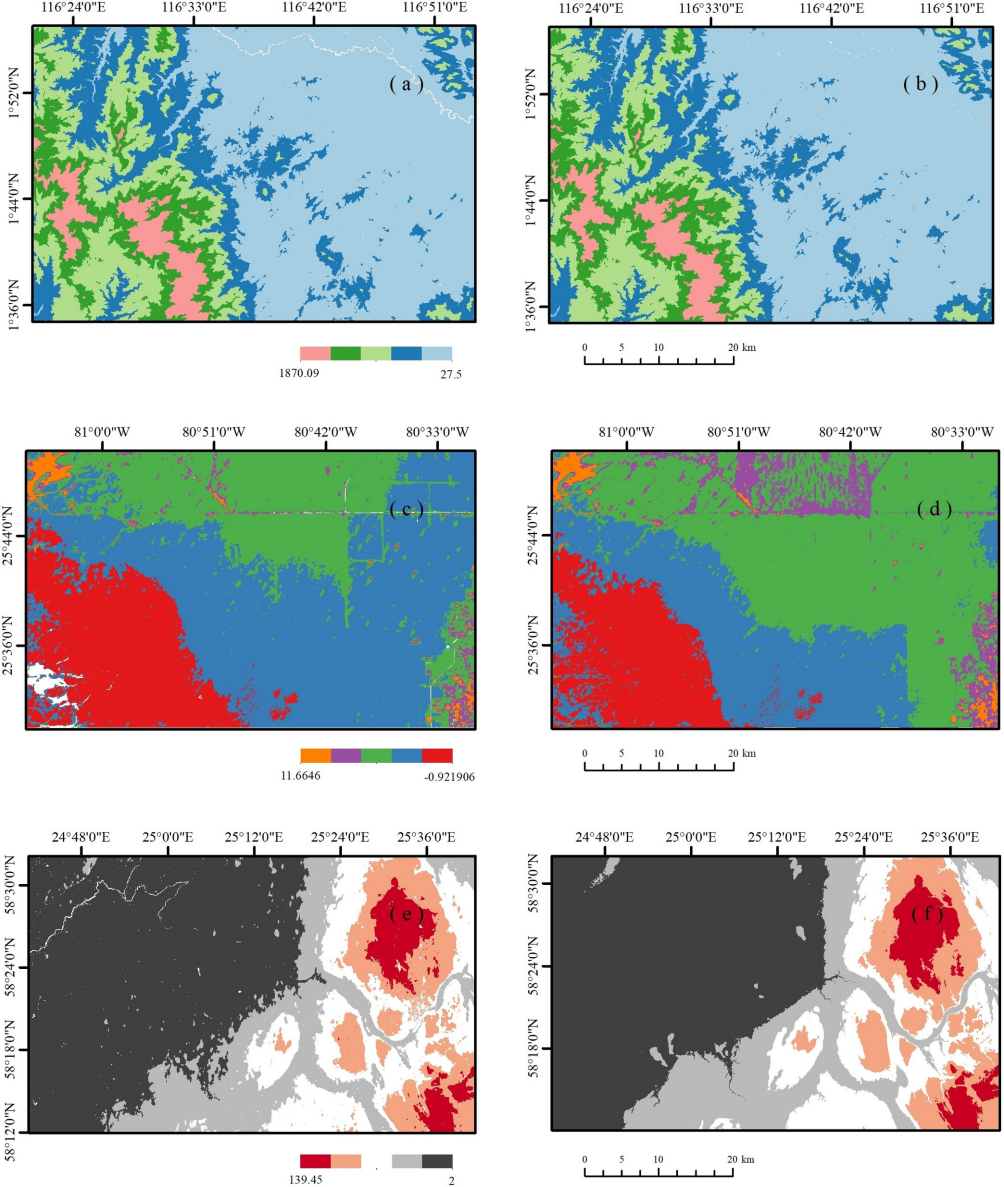
Locations of test sites. Created using public data from http://www.naturalearthdata.com/.

The current experimental area is geographically distributed across four different forest types. Area I is located in the northeastern part of Kalimantan Island, Indonesia (116°21′ to 116°54′ E, 1°35′ to 1°57′ N). The Kalimantan region is located on the Indonesian island of Borneo and is a tropical rainforest ecosystem [15]. The elevation range of the study area is from 89 to 1890 metres above sea level. The terrain is complex, with slopes ranging from 0° to 68°, showing diverse geomorphological patterns. Area II is located in the southern part of the state of Florida, USA (80°30′ to 81°6′ W longitude and 25°30′ to 25°51′ N latitude). The study area in Florida is a coniferous forest ecosystem [16]. It is located between -2 and 23 metres above sea level and is the flattest terrain of the four study areas. Slopes range from 0° to 16°, and the terrain is relatively gentle. Area III is located in western California, USA (120°12′ to 121° W, 39° to 39°22′ N). The study area in California is a mixed coniferous broadleaf forest ecosystem that ranges in elevation from 237 to 2740 m and has the steepest and most complex terrain [17]. Slopes range from 0° to 75°, and the terrain is varied. The IV area is located in the south-west of Pärnumaa, Estonia (24°42′ to 25°42′ E, 58°12′ to 58°33′ N). The study area in Pärnumaa belongs to the broad-leaved forest ecosystem. The area ranges in elevation from 2 to 152 metres above sea level and the terrain is relatively flat [18]. Slopes range from 0° to 31° and the terrain is relatively flat. The exact location of the study area is shown in **Fig 1**.

### Data preparations

#### ICESat-2 data

**Fig 2:**
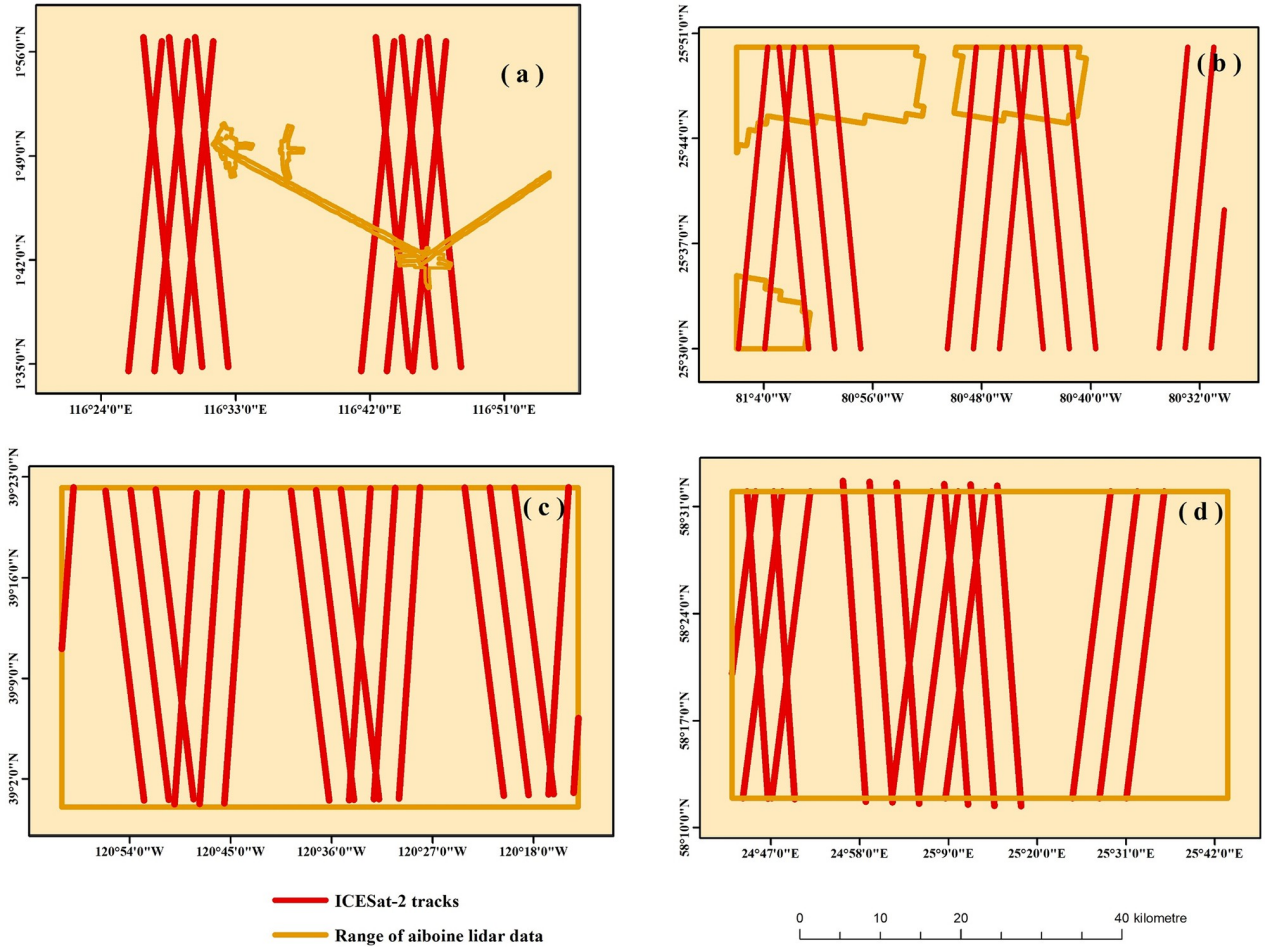
(a) Kalimantan region of Indonesia; (b) Florida, USA; (c) California, USA; (d) Pärnumaa region of Estonia. Created using public data from http://www.naturalearthdata.com/.

In 2018, NASA launched ICESat-2, the follow-up mission to the original ICESat, implementing technological upgrades. The data from ICESat-2 overcame the limitation of low along-track resolution observed in the previous ICESat mission. Among the 22 standard products generated by ICESat-2, the class 3A surface-specific data products include glacier and ice-cap heights, sea-ice drywall, vegetation canopy heights, ocean-surface topography and inland water body heights. Of all these Level 3A data products, the ATL08 product refers to the valuable topography and canopy height information extracted from ICESat-2’s 2A product ATL03, which is a terrestrial vegetation data product. Also, the data include a series of relevant descriptive parameters such as the signal-to-noise ratio (used to describe the ratio of signal strength relative to noise. A higher signal-to-noise ratio indicates a better quality signal with less interference from noise), ascent/descent trajectories and cloud cover. These descriptive parameters can be used to select sampling points for ATL08 products with higher accuracy. In subsequent sections of this document, ICESat-2 refers exclusively to its Class 3A ATL08 product. Recent studies have validated the sub-canopy terrain accuracy of ATL08 products using airborne lidar data [19, 20], demonstrating the ability of the terrain results generated by ICESat-2 ATL08 products to correct the elevation errors in forested areas of SRTM DEM. The spatial distribution of the training data tracks is illustrated by the solid red line in **Fig 2**, and specific track information is detailed in **Table 1**.

**Table 1:** Details of ICESat-2 used in the training data in this study.

Study area	ICESat-2 granules	Study area	ICESat-2 granules
Kalimantan	20220127222545_05601401_006_01 20220213093230_08111407_006_01 20220225210150_10021401_006_01 20220515051227_08111507_006_01	Florida	20220110003025_02861407_006_01
			20220127112543_05531401_006_01
			20220207230626_07281407_006_01
			20220312213412_12311407_006_01
			20220330082933_01111501_006_01
California	20220111030451_03031406_006_01	Pärnumaa	20220109050557_02741402_006_01
	20220112144900_03261402_006_01		20220204162327_06781406_006_01
	20220209014051_07451406_006_01		20220207034158_07161402_006_01
	20220210132500_07681402_006_01		20220305145932_11201406_006_01
	20220310001657_11871406_006_01		20220308021804_11581402_006_01
	20220315115247_12711402_006_01		20220403133536_01751506_006_02

#### DEM data

Copernicus DEM is derived from the TanDEM-X mission provided by the European Space Agency (ESA). There are three versions of Copernicus DEM data: EEA-10 (0.4 arc-seconds), GLO-30 (1 arc-second), and GLO-90 (3 arc-seconds). In this study, the 1 arc-second (30m) version (ESA, 2021) was chosen. COP30DEM is considered an ideal choice, with several advantages. Firstly, this dataset is allowed for any purpose, providing users with greater flexibility and freedom. Secondly, unlike the striping noise commonly found in SRTM data, COP30DEM is not affected by this noise, providing clearer and more reliable terrain height information. COP30DEM smooths out the noise effectively, enhancing the elevation signal and further improving the accuracy and usability of the data [21]. Finally, COP30DEM is a global dataset with extensive coverage, enabling its application worldwide. Considering these features, COP30DEM is the preferred option for generating forest DEM data with its multiple advantages of flexibility, clarity, accuracy, and global coverage.

The point density of airborne LiDAR data for reference terrain height in Kalimantan, Indonesia ranges from 4 to 10 p/m2. The corresponding 1m resolution digital terrain model (DTM) was downloaded from the Oak Ridge National Laboratory Distributed Active Archive Center. The point density of airborne LiDAR data for reference terrain height in Estonia is approximately 18 p/m2. The corresponding 1m resolution DTM was downloaded from the Estonian Land Board. The point density of airborne LiDAR data for reference terrain height in Florida and California ranges from 12.5 to 17.3 p/m2. The corresponding 1m resolution DTM was downloaded from the 3D Elevation Program of the U.S. Geological Survey (USGS). The spatial extent of the airborne LiDAR data is represented by the orange polygons in **Fig 2**.

#### Other data

Based on previous studies, it has been found that the vertical error of digital elevation models is closely related to the morphological attributes of the terrain surface. Research indicates that the primary factors influencing DEM accuracy include slope [22], surface segmentation depth [23], and relief amplitude [24]. To improve accuracy, researchers chose data from Landsat-9, as differences between bands can be used to delineate forest types, which are also important factors affecting DEM accuracy [25]. Landsat-9 observations may be affected by radiative transfer from surface targets in the visible and infrared bands due to brightness variations caused by clouds and cloud shadows. To reduce this effect, images with Cloud Cover Range below 6% were selected for this study. Given the possibility of noise in COP30DEM, image processing operators were employed for processing. For high-frequency noise, Gaussian differencing functions were applied with different parameter values, generating three sets of images: Gauss_1, Gauss_2, and Gauss_3. By choosing different combinations of σ values (e.g., σ1 and σ2), different image processing effects are achieved. The combination of σ1 = 0.5 and σ2 = 1 detects fine edges and features; σ1 = 1 and σ2 = 2 strikes a balance between detail preservation and noise smoothing; and σ1 = 2 and σ2 = 4 strongly smoothes out the noise and preserves large-scale features. Additionally, Sobel operators were utilized for edge detection to further enhance the quality of DEM data.

The training and prediction data for this experiment consist of 23 explanatory variables used to forecast the DEM data in densely forested mountainous areas. The specific data sources and catalogue are listed in **Table 2**.

**Table 2:** Data used in this study.

Classes	Data	Resolution	Reference	Usage
DEM	COP30DEM	30 m	https://panda.copernicus.eu/panda	Original data for correction、Inputs for terrain attribute calculations
Landsat	Lansat-9 (11 bands)	15, 30, 90 m	https://glovis.usgs.gov/app	For geographic alignment
Canopy Height	Canopy Nico	10 m	https://www.research-collection.ethz.ch/handle/20.500.11850/609802	Covariates used for prediction
Vegetation Cover	Treecover2010	30 m	https://glad.umd.edu/dataset/global-2010-tree-cover-30-m	Covariates used for prediction
Land Use Classification	Sentinel-2 Land Use/Land Cover	10 m	https://esri.maps.arcgis.com/apps/instant/media/index.html?appid=fc92d38533d440078f17678ebc20e8e2	For surface feature identification
Terrain Attribute	Slope, Surface Cutting Depth, Relief amplitude, Terrain roughness	30 m	Calculated by COP30DEM	For elevation reference correction
Denoising	Gauss_1, Gauss_2, Gauss_3, Sobel	30 m	Calculated by COP30DEM	For edge detection and noise smoothing

## Methodologies

Due to the inability of ICESat-2 to provide radar altimetry equivalent spatial coverage, these data cannot be used independently to generate medium-resolution (30~100 meters) digital elevation models. This paper combined freely available DEM products with ICESat-2 elevation data to establish a vegetation correction model specifically for COP30DEM at selected study locations to meet global requirements. In this study, ground elevations in forested areas were obtained by employing a hybrid algorithm that used ICESat-2 provided ground heights as ground truth to regressively correct the COP30DEM elevation data, resulting in ground elevations for forested regions. The results obtained from various models were compared, and the forest DEM generated by the model with the best performance was selected, followed by a comparison with other accessible forest DEMs.

### Data preprocessing

Considering the overall objective of model-based correction for the elevation of COP30DEM in forested areas, the experiment utilized photons labeled as ground in ATL08. In vegetated areas, the complete vegetation and terrain information is difficult to record for weak beams due to the shielding effect of the canopy. Therefore, it is necessary to exclude the ICESat-2 ATL08 products obtained from weak beams based on the parameter description of satellite ascending and descending orbits. Additionally, clouds and fog can also affect the accuracy of the terrain information provided by ICESat-2 ATL08 products, thus terrain data with cloud layer flags should be excluded.

First, convert the elevation values of ICESat-2 to the EGM2008 vertical datum, then match the trajectory of ICESat-2 with COP30DEM based on the closest geodesic distance (Geodesic distances are calculated between two points on the Earth’s surface by measuring the shortest path between them. This method takes into account the curvature of the Earth and is therefore generally more accurate than straight-line distances measured on a flat surface.). Establish a one-to-one correspondence between the point data of ICESat-2 and COP30DEM, and for the matched data, identify ATL08 point data with height differences exceeding the threshold as outliers and remove them. The thresholds set for this study are -10 meters and 50 meters. Several reasons justify selecting data within this height range. First, including negative elevations (-10 meters) helps address any potential negative bias present in the COP30DEM data. Second, setting an upper limit of 50 meters corrects significant positive biases introduced by canopy height.

To mitigate potential errors arising from positional deviations caused by factors such as refraction of ICESat-2 photons in water, Land Use/Land Cover (LULC) data derived from Sentinel-2 imagery was employed to mask water bodies in COP30DEM data, thereby delineating land areas.

Considering the footprint diameter of ICESat-2 is 17 m and the resolution of ATL08 is 100 m, when preparing the feature values, it is necessary to obtain the elevation values of ICESat-2 corresponding to COP30DEM points and include the elevation values of the eight neighboring pixels around that position. Subsequently, the COP30DEM is resampled to 90m and the same operation as the COP30DEM is performed to obtain the elevation values of the corresponding position and the 8 neighbouring image elements around it. Through this process, a total of 18 elevation feature values including the target point were obtained. The training and prediction data for the other explanatory variables used in this experiment were resampled to 30m to match the COP30DEM to enable pixel-by-pixel corrections.

### Algorithm interpretation

#### FA and SSA algorithm

The Firefly Algorithm (FA) and the Sparrow Search Algorithm (SSA) are two nature-inspired metaheuristic optimization algorithms. The FA simulates the flashing behavior of fireflies and optimizes the objective function using brightness to represent attraction. However, the FA suffers from premature convergence and is prone to getting trapped in local optima when searching for the target in the solution space. The SSA is derived from the foraging and predation behavior of sparrows, exhibiting advantages such as improved search accuracy and faster convergence. Nevertheless, the population diversity of SSA gradually decreases in later iterations, leading to a tendency to converge to local optima. To overcome the limitations of these algorithms, this study proposes an improved SSA search algorithm that incorporates the ingestion strategy of the FA algorithm. This improvement aims to enhance the solution diversity and global search capability to improve the accuracy of the method and is referred to as the Firefly Algorithm-based Sparrow Search Optimisation Algorithm (FA-SSA algorithm).

#### LightGBM framework

LightGBM and the random forest model widely used in remote sensing differ in several key aspects. LightGBM adopts a method of sequential tree rebuilding, emphasizing the use of a leaf-based tree growth framework for correction. In contrast, random forests achieve this by creating multiple independent decision trees, each using random features and sample subsets. During the training process, LightGBM uses gradient boosting and dynamically adjusts loss function parameters during each iteration, while random forests independently train each tree and use random samples. The efficiency of LightGBM comes from its leaf growth strategy and histogram linking method, making it faster than random forests. However, compared to random forests, LightGBM’s sequential tree construction makes feature importance analysis more complex, as random forests provide better interpretability through individual decision trees. In this study, the LightGBM regression model is adopted as the main prediction tool. The leaf growth strategy and histogram linking method of LightGBM effectively reduce the memory occupation and make full use of the data for training while ensuring the speed. Combining the feature extraction capability of CNN model and the prediction capability of LightGBM further improves the accuracy of forest DEM correction.

#### Model building and prediction

**Fig 3:**
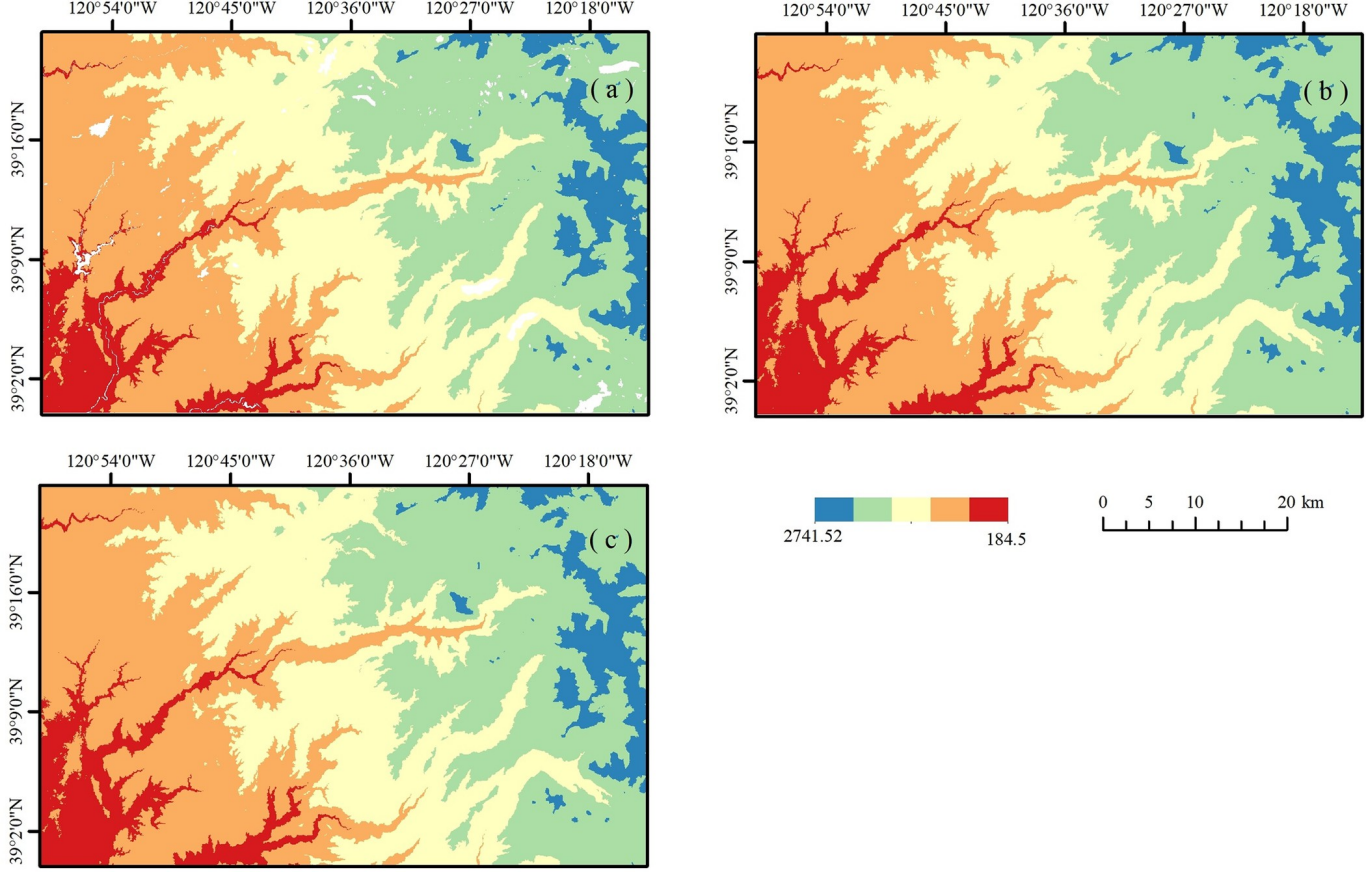
Workflow of COP30DEM deviation correction model.

Certain single algorithms, such as FA or SSA, are prone to falling into local optima within the solution space, leading to premature convergence or performance degradation of the algorithm [26]. Furthermore, the performance of a single model can be susceptible to data noise or specific distributions of the training set due to the involvement of a large number of feature values in the data training and prediction processes, which can lead to performance fluctuations of the model. Ensemble learning can alleviate such fluctuations by averaging the predictions of multiple models [27]. Therefore, the FA-SSA-CNN-LightGBM model was constructed for DEM correction. The processing procedure is shown in **Fig 3**. The specific steps are as follows:

1. Organize the preprocessed data by removing missing values and duplicates, ensuring coverage of data from different study areas. Integrate all the data into one dataset and randomly select 70% of the data as the training set, while the remaining 30% is used as the test data to validate the model. This distribution ratio has been proven to yield optimal modeling results in scenarios such as groundwater potential mapping and landslide analysis [28, 29].
2. Use FA-SSA to perform noise reduction and feature extraction on the original data. FA-SSA extracts significant feature components from the original data through iterative optimization and singular spectrum analysis. These extracted features are then input into a CNN (Convolutional Neural Network) for further feature learning. CNN can effectively capture local patterns and complex features in data. Finally, the high-level features extracted by CNN are input into LightGBM for the final regression task. As an efficient gradient boosted decision tree algorithm, LightGBM can handle large-scale data and provide good prediction performance.
3. Construct the FA-SSA-CNN-LightGBM prediction model to reveal the relationship between DEM and the model’s input variables using CNN-LightGBM. The firefly algorithm (FA) and sparrow search algorithm (SSA) are combined in a sequential manner to jointly optimize the hyperparameters of CNN-LightGBM and determine the initialization parameter settings for the FA-SSA-CNN-LightGBM prediction model. Where Learning Rate is set to 0.0001, Number of Hidden Layers is set to 3, and Max Depth is set to 10.
4. Train the FA-SSA-CNN-LightGBM prediction model using the training set and evaluate its predictive performance on the test set. Performance validation and evaluation of the trained FA-SSA-CNN-LightGBM prediction model are conducted using metrics such as correlation coefficient R2, mean absolute error (MAE) and mean squared error (MSE). In order to better demonstrate the effectiveness of the different methods, a comparison of the experimental results of the following methods was carried out:
  - Methods using only CNN-LightGBM
  - Combining FA or SSA with CNN-LightGBM approach
  - Combining FA-SSA with CNN-LightGBM approach
5. The COP30DEM of the study area was predicted using the completed trained model to obtain the forest DEM.

### Accuracy assessment

To evaluate the corrected COP30DEM, the elevation bias between it and the reference LiDAR DEM was calculated. The accuracy of the corrected COP30DEM was evaluated by calculating three accuracy statistics: mean error (ME), mean absolute error (MAE), and root mean square error (RMSE).

## Results

### Model validation

#### ICESat-2 accuracy verification

Considering that ICESat-2 terrain height serves as the foundation of the COP30DEM correction model, a direct comparison was conducted between airborne LiDAR and ICESat-2 ATL08 terrain photons to evaluate the vertical height differences between the two. **Table 3** presents the mean height bias and root mean square error of ICESat-2 relative to the airborne data within each study area. Overall, the mean absolute error (RMSE, a statistical measure that indicates the average magnitude of the difference between predicted values and actual values) of ICESat-2 elevation was 0.08 m, and the RMSE was 53 cm.

**Table 3:** Error of ICESat-2 with respect to airborne data.

Region	Kalimantan			Florida			California			Pärnumaa		
Error Metric	ME	MAE	RMSE	ME	MAE	RMSE	ME	MAE	RMSE	ME	MAE	RMSE
ICESat-2	0.16	0.32	0.44	-0.13	0.42	0.49	0.02	0.47	0.55	-0.29	0.59	0.65

#### Comparative analysis of model advantages

**Fig 4:**
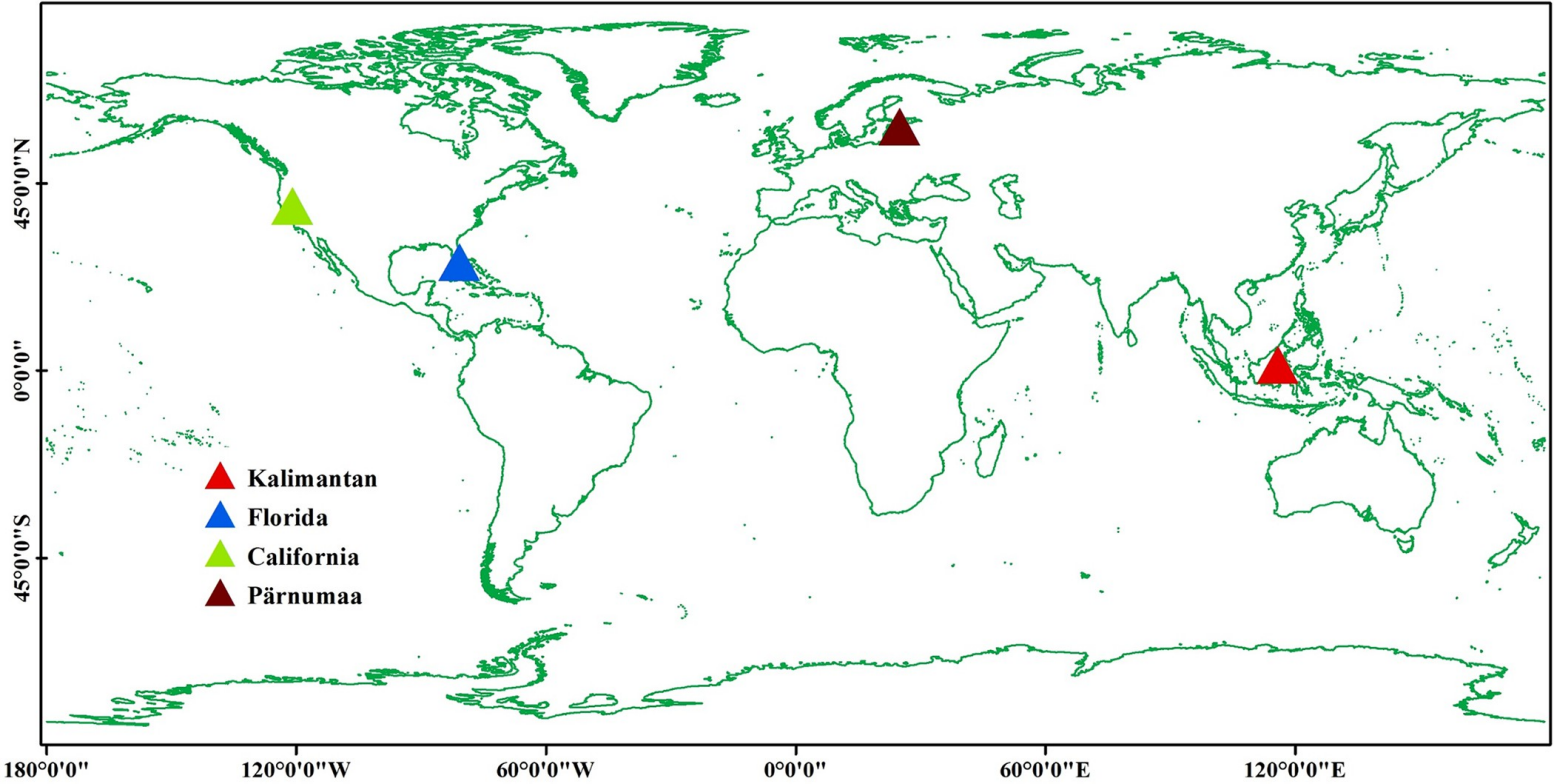
Prediction results of different models.

To validate the superiority of the CNN-LightGBM hybrid model in DEM correction compared to other models, comparative experiments were conducted with the FA-SSA-CNN-LightGBM model against the FA-SSA-LightGBM model, the FA-SSA-CNN-SVR model, and the FA-SSA-SVR model within the same sample space. Support Vector Regression (SVR) is a neural network model capable of finding optimal solutions and suppressing model complexity while maintaining learning capability. SVR has significant advantages in handling small samples, high samples, and nonlinear data, and exhibits great robustness and generalisation performance in solving regression. The results in **Fig 4** demonstrated that the FA-SSA-CNN-LightGBM model achieved the highest prediction accuracy, and its predicted results matched well with the actual values. The accuracy of FA-SSA-LightGBM is higher than FA-SSA-SVR, and the accuracy of FA-SSA-CNN-LightGBM is higher than FA-SSA-CNN-SVR, indicating that LightGBM is more suitable for forest DEM correction compared to SVR. It is worth noting that the inclusion of an attention mechanism in CNN leads to higher accuracy in FA-SSA-CNN-LightGBM compared to FA-SSA-LightGBM, and higher accuracy in FA-SSA-CNN-SVR compared to FA-SSA-SVR, highlighting the importance and necessity of attention mechanisms in CNN models. In this study, the CNN model employs a spatial attention mechanism, which helps the model to identify important terrain feature areas such as steep slopes and valleys. By increasing the weights of these regions, the model is able to correct the elevation error more accurately, thus improving the overall prediction accuracy.

Taking into account various factors, the CNN-LightGBM hybrid model was selected due to its predictive performance in the COP30DEM correction task. Therefore, the next section will focus on discussing the effect of optimization algorithms on the correction performance of the CNN-LightGBM hybrid model.

### Accuracy verification

#### Mixed model accuracy verification

In this paper, four models were established, including FA-SSA-CNN-LightGBM, SSA-CNN-LightGBM, FA-CNN-LightGBM, and CNN-LightGBM models, for correcting COP30DEM to evaluate the advantage of incorporating Firefly Search perturbed Sparrow Search Algorithm (FA-SSA) optimization algorithm. The accuracy of the predicted results of these models was verified using LiDAR DEM within the region. **Table 4** shows the ME, MAE, and RMSE of these models compared to LiDAR DEM. It can be seen that FA-SSA-CNN-LightGBM outperforms other algorithms in correcting COP30DEM, including lower MAE and RMSE, highlighting the outstanding ability of this model in capturing data patterns and accurate prediction.

**Table 4:** Error of models.

Error Metric	Model	Kalimantan	Florida	California	Pärnumaa
ME	FA-SSA-CNN-LightGBM	**-0.21**	**0.41**	0.23	**-0.05**
	FA-CNN-LightGBM	-1.92	-3.34	0.94	-1.46
	SSA-CNN-LightGBM	-1.16	-1.80	**-0.02**	-1.23
	CNN-LightGBM	-2.68	-3.88	1.88	-1.57
MAE	FA-SSA-CNN-LightGBM	**1.48**	**0.54**	**1.44**	**1.47**
	FA-CNN-LightGBM	2.98	3.35	2.44	2.65
	SSA-CNN-LightGBM	2.63	1.81	2.35	2.60
	CNN-LightGBM	3.50	3.88	2.84	2.69
RMSE	FA-SSA-CNN-LightGBM	**1.76**	**1.09**	**1.68**	**1.70**
	FA-CNN-LightGBM	3.57	3.78	3.03	3.16
	SSA-CNN-LightGBM	3.07	2.21	2.77	3.08
	CNN-LightGBM	4.24	4.36	3.61	3.21

In summary, through the comparison of various hybrid models in Section 4.1.2, it was found that the CNN-LightGBM model can predict more accurately. In terms of optimization algorithms, the Sparrow Search Optimization Algorithm with Firefly Search Perturbation (FA-SSA) can effectively improve the prediction results, with its accuracy far surpassing the other three scenarios. Therefore, based on the results analysis of these two sections, the FA-SSA-CNN-LightGBM model outperforms other models in terms of the coefficient of determination R2 and prediction accuracy.

For the sake of convenience, the DEM of forest areas generated by the FA-SSA-CNN-LightGBM model will be referred to as DMFDEM in the following. Next, a further comparison will be made between DMFDEM and other DEMs. This comparison aims to evaluate the relative advantages of DMFDEM in forest terrain modeling and further validate its feasibility in practical applications.

#### Calibration results and other DEM accuracy verifications

DMFDEM and FABDEM (Forest and Buildings removed Copernicus DEM), a similar dataset, were validated for accuracy with airborne LiDAR DEM data. The results indicate that DMFDEM exhibits higher accuracy compared to FABDEM. **Table 5** show the ME, MAE, and RMSE between the DEMs of different study areas and the airborne LiDAR DEM. While ME can be used to show the overall bias of the DEM, large positive and negative errors may cancel each other out, resulting in a low ME but high RMSE. Therefore, a comprehensive comparison of DEMs across metrics is crucial to avoid misinterpretation of DEM accuracy.

**Table 5:** DEM error verified by airborne data.

Region	Error Metric	DMFDEM	FABDEM	COP30DEM	GEDI
Kalimantan	ME	**-0.21**	0.39	25.82	-1.25
	MAE	**1.48**	1.73	25.82	2.17
	RMSE	**1.76**	2.78	26.39	3.61
Florida	ME	**0.41**	-0.89	5.49	-0.87
	MAE	**0.54**	1.35	5.63	0.96
	RMSE	**1.09**	1.89	6.45	1.40
California	ME	**0.23**	0.35	9.37	-0.98
	MAE	**1.44**	2.25	10.08	2.99
	RMSE	**1.68**	2.66	12.12	3.17
Pärnumaa	ME	-0.05	**-0.02**	6.19	0.79 *
	MAE	**1.47**	1.54	6.27	1.55 *
	RMSE	**1.70**	2.12	8.08	2.02 *

As GEDI can only acquire ground elevation data between 52°N and 52°S, the study area in Pärnumaa is near 58 degrees north latitude, leading to the utilization of EU DEM (European Digital Terrain Model). This dataset has a resolution of 30m, covering the western part of Europe, with a coordinate system of ETRS89-extended / LAEA Europe and a reference surface of EGM2008. In **Table 5**, values marked with ‘*’ represent EU DEM data.

When validated with airborne data, the RMSE of DMFDEM is less than 2 meters in all four regions, with the best performance reaching 1.09 meters and the minimum MAE at 0.54 meters. In three out of the four regions, the RMSE of DMFDEM is more than 0.4 meters lower than that of FABDEM, especially exceeding 1 meter in the Kalimantan region of Indonesia. Compared to GEDI, DMFDEM shows superior results, with the RMSE difference exceeding 1.4 meters in two regions. In the Pärnumaa region of Estonia, DMFDEM demonstrates advantages over EU DEM, with a significantly lower ME value, similar MAE, and RMSE showing similar performance to ME. In comparisons with other DEMs, DMFDEM consistently exhibits the lowest comprehensive error indicators.

FABDEM is a digital elevation model generated from airborne LiDAR data, which removes trees and buildings, and needs to consider both forested and built-up areas. In contrast, DMFDEM focuses solely on forested areas, thus exhibiting a clear advantage over FABDEM in forested regions. Although GEDI is designed for forested areas, both validation methods showed slightly lower accuracy compared to DMFDEM. The ground truth values used in this correction process were single-photon point cloud data from ICESat-2, whereas GEDI uses waveform data.

**Fig 5:**
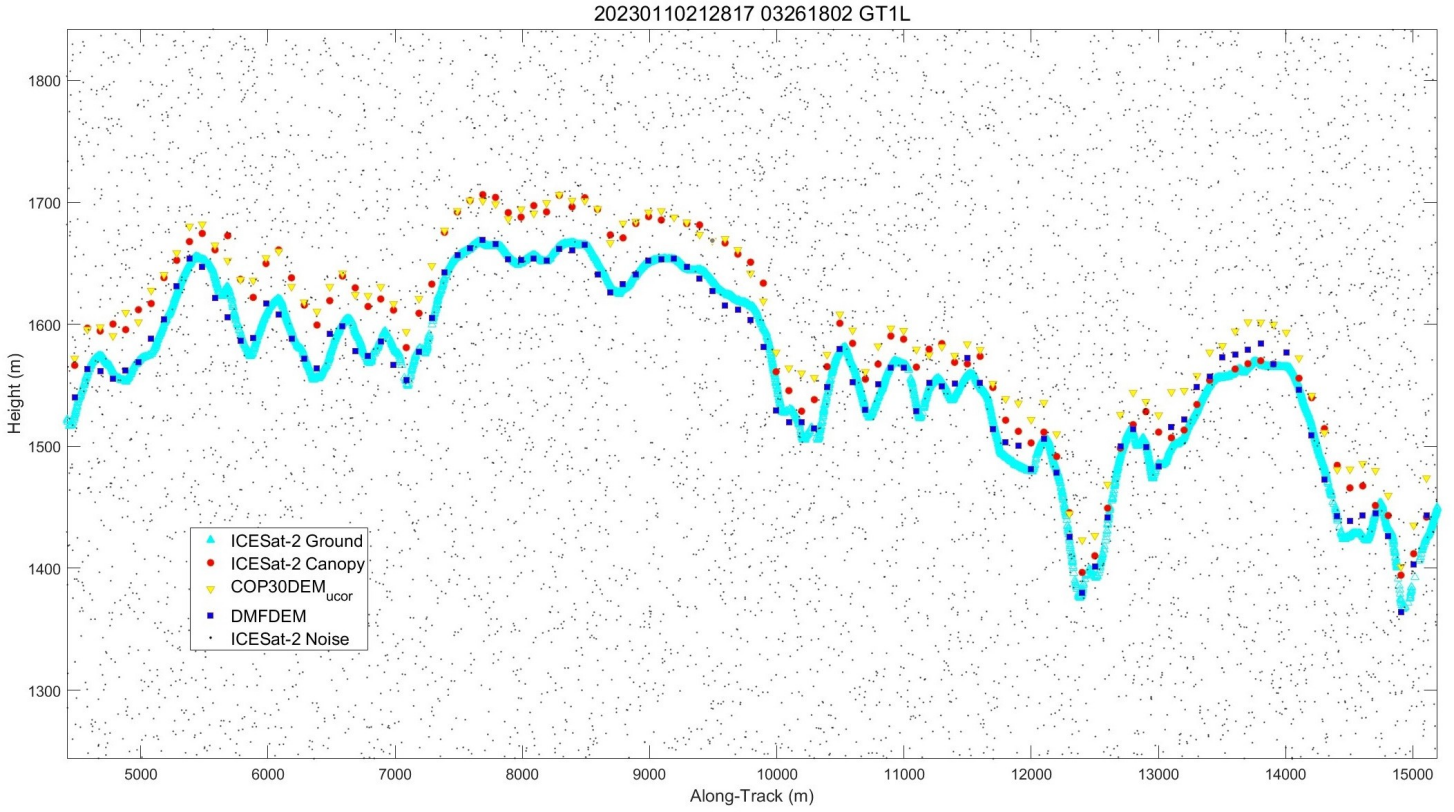
Histogram comparing DMFDEM, FABDEM, EU DEM against the validation data.

**Fig 5** depicts the error histograms drawn for Pärnumaa, where DMFDEM, FABDEM, and EU DEM generally follow a normal distribution. However, DMFDEM and FABDEM exhibit negative skewness, while EU DEM shows positive skewness. The centers of all DEMs are close to zero, but the area under the curve near zero is largest for DMFDEM. The distribution of DMFDEM and FABDEM has similar peaks, but due to FABDEM containing more negative errors, its distribution is flatter. EU DEM has the lowest peak, and its distribution is the flattest among the three. The shapes in **Fig 5** also confirm the error computation values in **Table 5**.

Paired-sample t-test (a statistical method used to compare whether the means of the same group of subjects or experimental units are significantly different under two different conditions) analyses were conducted at different locations to compare the performance of the three digital elevation models (DEMs), FABDEM, COP30DEM, and GEDI, relative to DMFDEM. The results in **Table 6** show that there is a significant difference in elevation errors between DEMs (FABDEM, COP30DEM, GEDI) and DMFDEM in all cases. Specifically, in the Pärnumaa region, the significant differences between FABDEM and DMFDEM are small, indicating that they perform similarly in this region; however, in the Kalimantan, Florida, and California regions, the differences in error between all three DEMs and DMFDEM are very significant, indicating that the model perform significantly better in these regions than in others. These differences may be due to the complexity of the terrain features in each region and the ability of the models to adapt to different terrains. For example, in the Pärnumaa region, FABDEM exhibits a similar level of error as DMFDEM, which may be due to the specific terrain features in this region being more compatible with the FABDEM generation algorithm. In other areas, FABDEM, COP30DEM, and GEDI failed to accurately capture the terrain features represented by DMFDEM, resulting in significant error differences.

**Table 6:** Paired t-test for detecting the difference between DMFDEM errors and other types of DEM errors.

	FABDEM	COP30DEM	GEDI
Kalimantan	**0.0**	**0.0**	**0.0**
Florida	**4.641×10** ^**−302**^	**0.0**	**0.0**
California	**5.560×10** ^**−15**^	**0.0**	**0.0**
Pärnumaa	**0.007**	**0.0**	0.0 *

Bold values are significant differences at P < 0.05

Although validation has shown that DMFDEM performs consistently and with high accuracy, it still faces some limitations. Firstly, despite the multiple advantages of the LiDAR DEM dataset, laser pulses cannot penetrate the tree canopy in areas with dense vegetation cover, affecting the accuracy of the ground model. In addition, data artefacts (e.g., voids, noise, etc.) are common, degrading visual quality and affecting subsequent data analysis. Similarly, when training the FA-SSA-CNN-LightGBM model, it is computationally expensive and prone to overfitting, despite its excellent performance in handling complex data and improving prediction accuracy. This requires a lot of computational resources and time, and if the training data is insufficient or the parameters are not properly chosen, the model may have poor generalisation ability on new data. Therefore, to cope with the limitations in the above, future research will focus on methodological improvements. Firstly, combining LiDAR, optical imagery and radar data to improve the accuracy of comparative DEMs for areas with dense vegetation cover. Second, developing algorithms to automatically identify and repair data voids and noise, and reducing artefacts through multiple data coverage fusion. For FA-SSA-CNN-LightGBM model optimisation, parallel and distributed computing techniques are used to improve efficiency and reduce costs; increasing the amount of data, using regularisation and cross-validation to improve model generalisation. Through these measures to ensure its reliability and validity.

### Visual comparison

**Fig 6:**
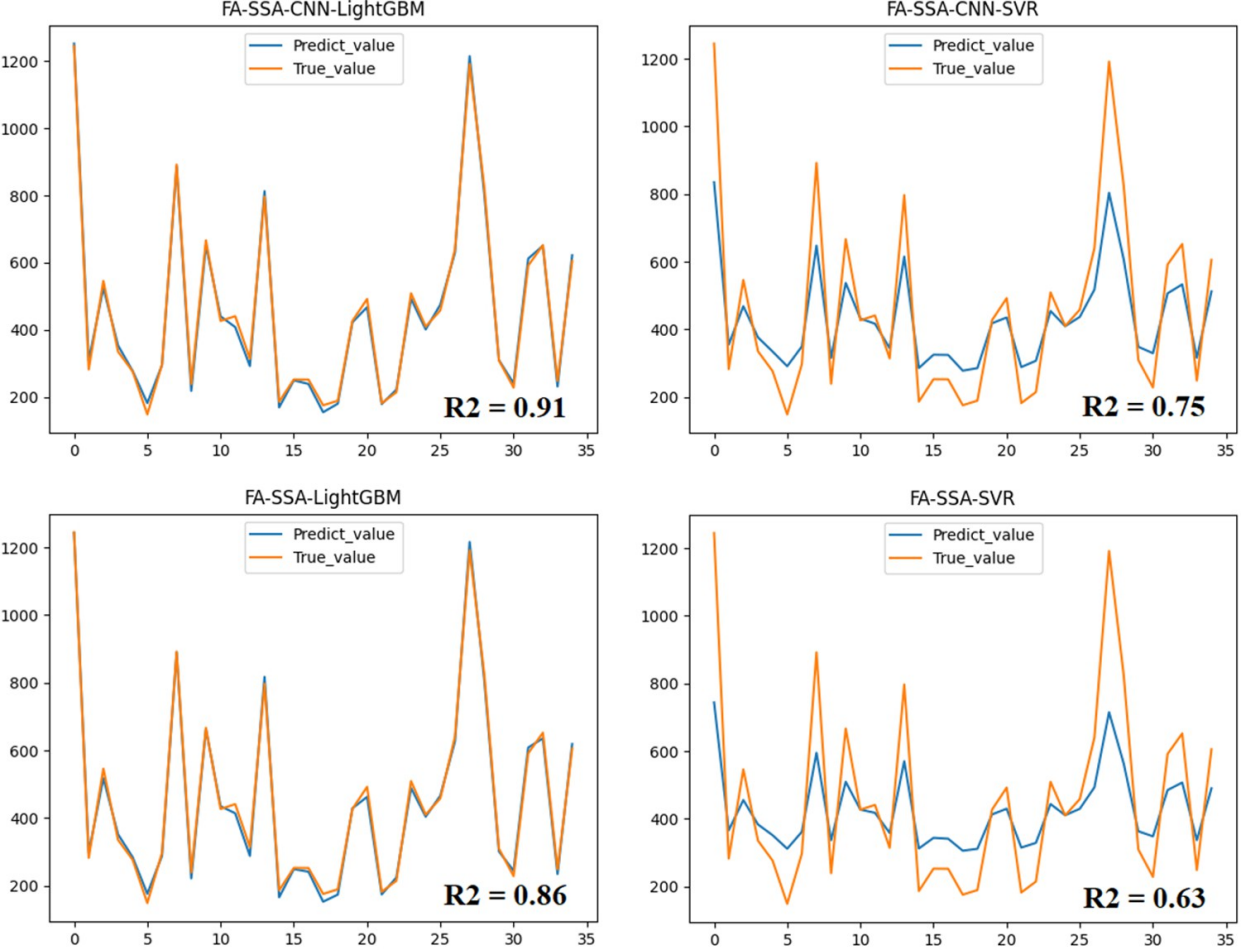
DEM in California (a) DMFDEM; (b) LiDAR DEM; (c) FABDEM.

**Fig 7:**
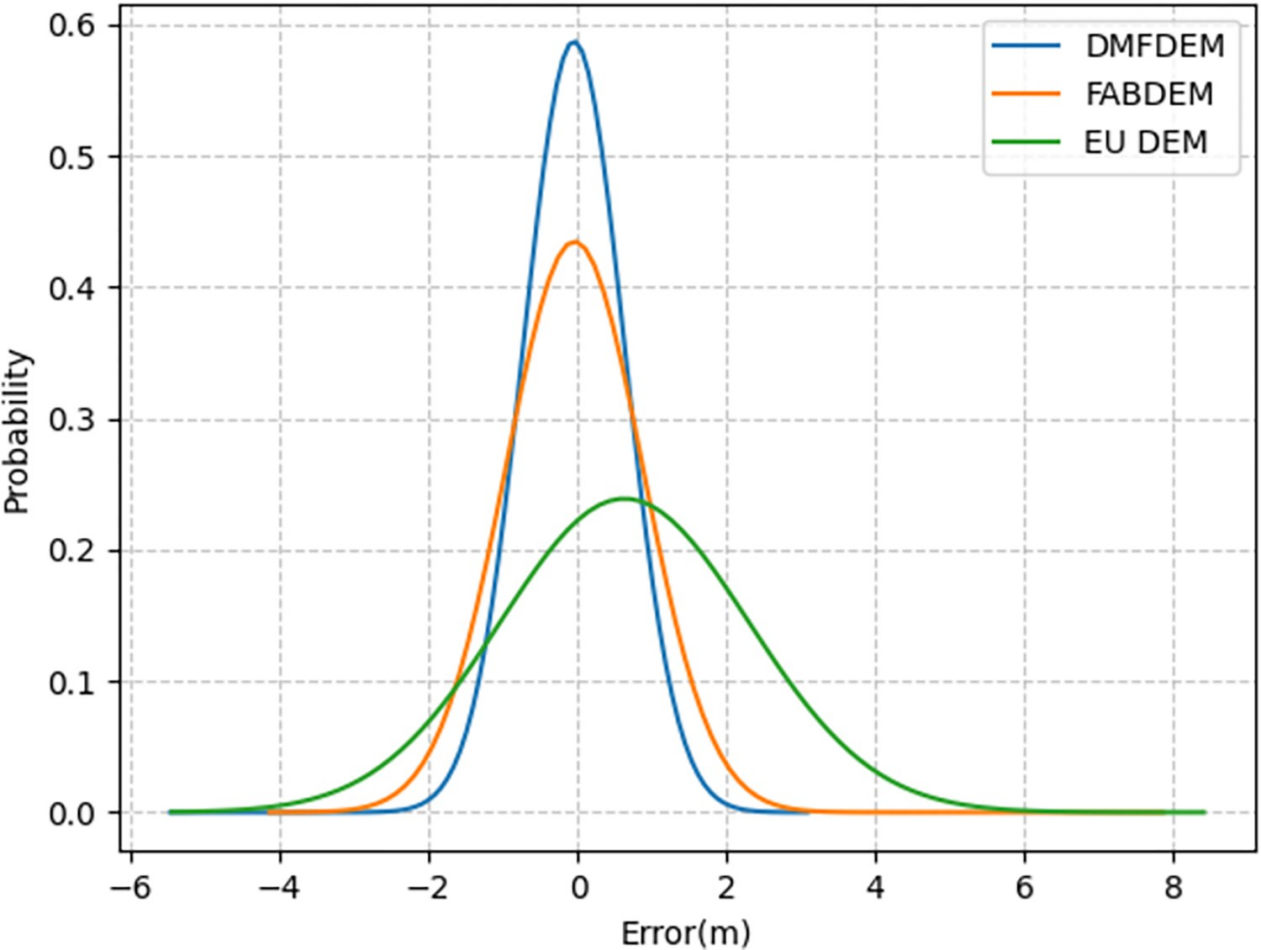
(a) DMFDEM in Kalimantan; (b) FABDEM in Kalimantan; (c) DMFDEM in Florida; (d) FABDEM in Florida; (e) DMFDEM in Pärnumaa; (f) FABDEM in Pärnumaa.

**Fig 6** presents the visualizations of DMFDEM, FABDEM, and LiDAR DEM in the California region. Due to the removal of water bodies in DMFDEM, there are certain blank areas in the image. A visual comparison of these three images indicates that the elevation of DMFDEM is very similar to LiDAR DEM, showing a good consistency, while FABDEM deviates from and mostly exceeds the elevation of LiDAR DEM. In detail, FABDEM fails to accurately represent details in certain areas, especially where there are significant changes in elevation, whereas DMFDEM performs better in capturing details compared to LiDAR DEM. **Fig 7** depicts visual comparisons of DMFDEM and FABDEM in the remaining three regions. Possible reasons for the low accuracy of FABDEM include the lack of accuracy of the vegetation removal algorithm in some areas, the possible introduction of errors in the interpolation method, and the effect of topographic variations that may result from different periods of data collection.

**Fig 8:**
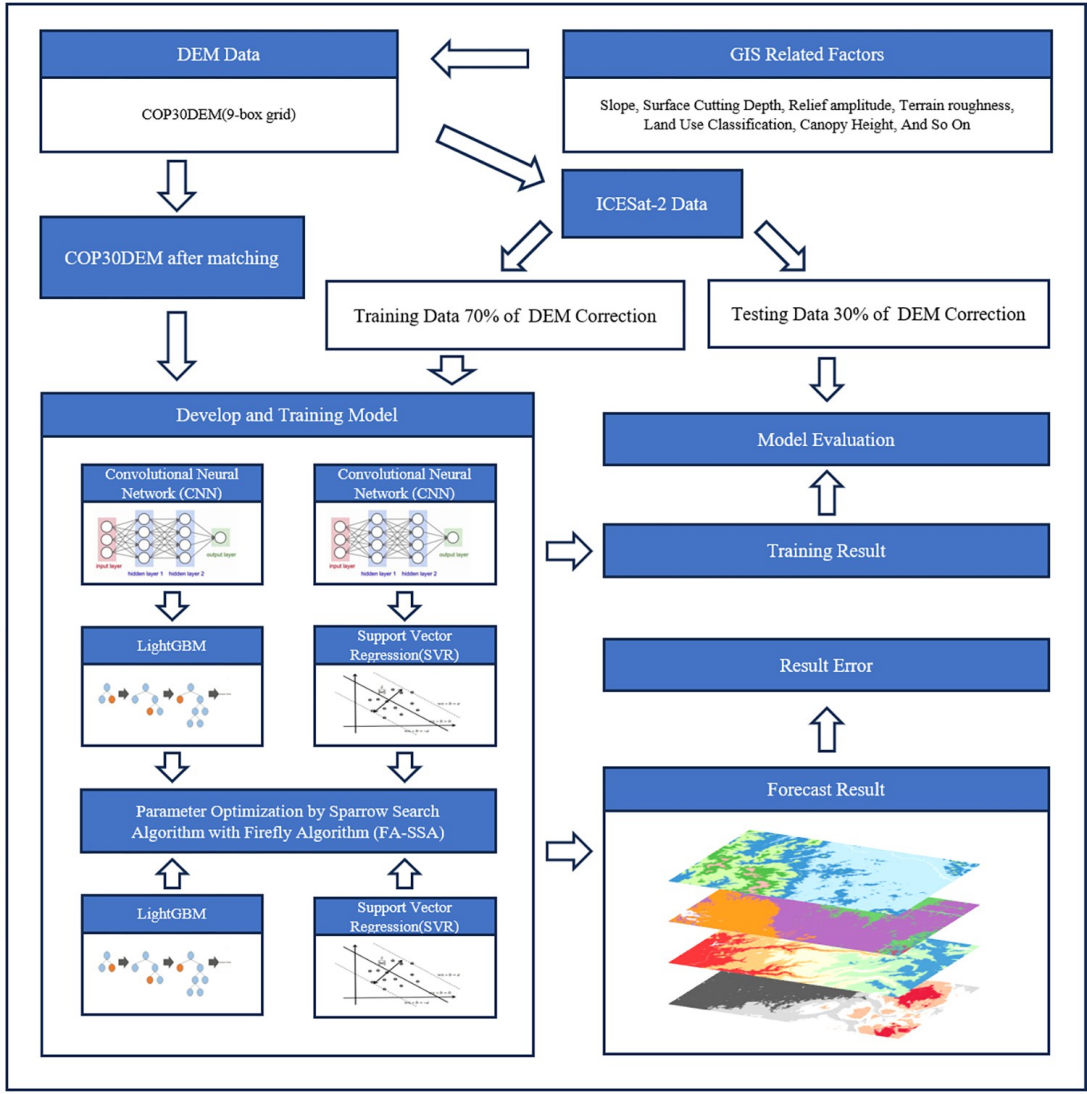
Transect in parts of California.

To provide a more intuitive presentation of the results of DMFDEM, **Fig 8** serves as an inset illustrating the height correction results by displaying a small portion of the ICESat-2 track in the California region, showing the original heights and the corrected heights. Through this figure, it is evident that DMFDEM can effectively fit the ground points of ICESat-2, indicating the excellent capability of FA-SSA-CNN-LightGBM in correcting the DEM in forested areas generated by COP30DEM. This highlights both the deficiencies in the original data and the practical utility of the developed model. Thus, DMFDEM will enhance the need for high-precision topographic information in different types of forest areas, including applications and models for ecological modelling, forest resource management and flood inundation modelling.

## Discussion

To address the limitation of incomplete spatial coverage of ICESat-2, this study developed a dedicated correction model for COP30DEM by integrating globally free DEM products and ICESat-2 elevation data. This paper focused on the ground elevation in densely forested areas, aiming to obtain more accurate ground elevation data using the FA-SSA-CNN-LightGBM hybrid model. Meanwhile, several other hybrid models were constructed as controls. These models utilized the ground elevation provided by ICESat-2 as the true value and performed regression correction on the COP30DEM elevation data to obtain more precise ground elevation information in forest areas. Accurate forest digital elevation models (DEMs) are critical in ecological modelling, carbon fixation estimation, and forest resource management. DEMs provide accurate topographic data to support habitat and species distribution modelling, estimation of carbon stocks and biomass, and hydrological modelling and flood risk assessment, which provide the underlying data for decision-making in forest management and environmental protection. Preliminary results show that the use of hybrid learning models is superior to other DEMs in terms of advanced correction in the field of DEM and accuracy in generating products, which highlights the importance of this study. The FA-SSA-CNN-LightGBM hybrid model integrates the advantages of feature selection, convolutional neural networks and lightweight gradient boosting compared to other models, which significantly improves the processing of complex data and the accuracy of prediction of complex data and prediction accuracy. However, such models are computationally expensive and prone to overfitting, requiring significant computational resources and time for effective training. To address these challenges, future research will focus on parallel computing and data augmentation techniques to improve the efficiency of the model and optimise the generalisation capability. There are also some limitations to this study. First, the number of study sites is limited and may not be representative. Second, fewer forest types were selected and the results may not be applicable to all forest environments. In addition, the LiDAR DEM comparison dataset has a concentrated and non-uniform distribution, limiting the accuracy and reliability of the study. These limitations need to be considered in further studies.

In the field of machine learning, ensemble learning integrates multiple basic models into the same framework to obtain a more powerful model with better performance than a single model [30]. On the other hand, deep learning architectures have diversity and can handle complex problems by automatically extracting features. However, the main challenge of deep learning is that tuning the optimal hyperparameters requires a lot of expertise and experience, which makes it a tedious and time-consuming task. To overcome this challenge, numerous research works have combined ensemble learning with deep learning in recent years [31–33]. In terms of DEM correction, Meadows and Wilson used random forest (RF), densely connected neural network (DCN) and fully convolutional neural network (FCN) techniques for bias correction of river depth measurements [34]. Kasi et al. applied machine learning methods such as genetic programming (GP) and artificial neural networks (ANN) to generate DEMs with higher vertical accuracy [35]. Dominick et al. and Kulp et al. respectively used LightGBM and neural network models to improve the accuracy of coastal DEMs, and employed multiple variables to train and predict the models [36, 37]. These research results demonstrate the feasibility of using machine learning-based methods combined with multiple variables to improve the accuracy of DEMs, providing important references for this study.

To assess the significant factors influencing the performance of the calibration model, RMSE was calculated for DMFDEM in California according to slope and vegetation coverage. The reason for choosing this region is that it exhibits a wide range of slope and vegetation cover, making the results more pronounced. The results are shown in **Table 7**. Under the same vegetation cover, as the slope increases, the RMSE gradually increases, consistent with previous research findings [38, 39]. This observation is also related to ICESat-2, as its error increases with the increase in terrain slope, as demonstrated by Zhu et al [11]. This indicates poorer predictive performance of the model under larger slope conditions, possibly due to the increased terrain complexity that makes the predictions more challenging. With increasing vegetation cover, the RMSE values mostly increase, occasionally decrease, depending on the specific slope range. This corroborates the findings of Lori et al [40]. and Mikhail et al [41]. In some cases, excessively dense vegetation may obstruct sensor observations. Additionally, slope has a more significant impact on accuracy, especially in steep slopes. Although changes in vegetation cover have a smaller impact on accuracy, they are still an important factor, particularly in areas with larger slopes where vegetation factors need to be carefully considered. Future research will incorporate additional environmental parameters into the model, not just the aforementioned canopy cover and slope features, as input components of the regression technique, and future work should focus on applying the correction technique to other regions of interest and vegetation morphologies. The use of ICESat-2 opens up the opportunity to apply the model to COP30DEM elevation products on a global scale, while also providing the possibility of using ICESat-2 data together with other DEM products beyond the COP30DEM data used here.

**Table 7:** RMSE was calculated according to slope and vegetation cover.

Slope Veg	0~10	10~20	20~30	> 30
0~25	0.95	1.31	1.66	2.19
25~50	1.15	1.38	1.73	2.11
50~75	1.29	1.53	1.78	2.06
75~100	1.71	1.90	2.04	2.20

RMSE in metres

## Conclusions

High-precision Digital Elevation Models play a crucial role in environmental and ecological analyses. However, most regions globally lack LiDAR DEM data, with currently available data mainly consisting of Digital Surface Models. Therefore, improving the accuracy of DEMs becomes particularly important. This study aims to use a hybrid learning model to calibrate the forest area Digital Elevation Model generated by COP30 DEM. To validate the accuracy of ICESat-2 data, its heights were compared with airborne LiDAR data. Additionally, the vertical errors of COP30 DEM data before and after calibration were evaluated. Compared to other global DEMs, DMFDEM exhibits higher accuracy across different regions and terrains. In four study areas, its RMSE is less than 1.7 meters, and the ME is close to 0, indicating minimal error in DMFDEM. The results demonstrate that this method significantly improves the accuracy of forest area DEMs, laying the foundation for applying this technology to further research on ecosystem and vegetation morphology changes. Furthermore, the calibration model approach demonstrates potential for correcting DEM products. These findings are of significant importance in advancing terrain mapping applications and assessment technologies, especially with the continuous increase in the availability of global observational data due to the introduction of new sensors.

## Supporting information

S1 Appendix Data sources. (DOCX)

S1 Table DEM error verified by ICESat-2. Table 1 lists the mean error (ME), mean absolute error (MAE), and root mean square error (RMSE) of different digital elevation models (DEMs) validated using ICESat-2 data from January to March 2023.
(DOCX)

S2 Table Training data and true values. Table 2 shows the datasets used for model training, where ICESat-2 data are considered true values and the rest of the data are training variables.
(DOCX)
