# Correction: LightGBM hybrid model based DEM correction for forested areas

**DOI:** 10.1371/journal.pone.0320535

**Published:** 2025-03-12

**Authors:** Qinghua Li, Dong Wang, Fengying Liu, Jiachen Yu, Zheng Jia

The images for Figs 1, 3–8 are incorrectly switched. The image that appears as Fig 1 should be Fig 7, the image that appears as Fig 3 should be Fig 6, the image that appears as Fig 4 should be Fig 1, the image that appears as Fig 5 should be Fig 8, the image that appears as Fig 6 should be Fig 4, the image that appears as Fig 7 should be Fig 5 and the image that appears as Fig 8 should be Fig 3. The figure captions appear in the correct order.

**Fig 1:**
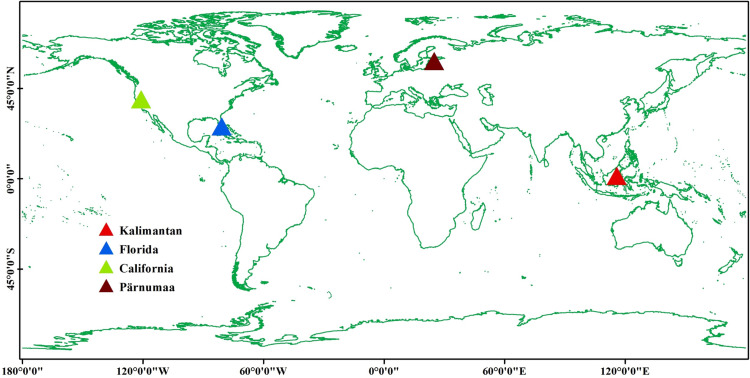
Locations of test sites. Created using public data from http://www.naturalearthdata.com/.

**Fig 3:**
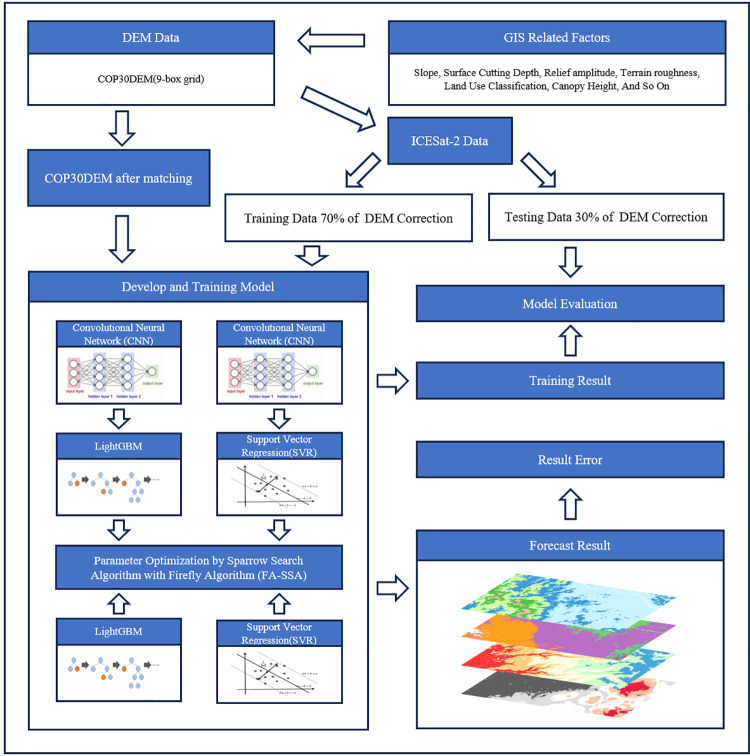
Workflow of COP30DEM deviation correction model.

**Fig 4:**
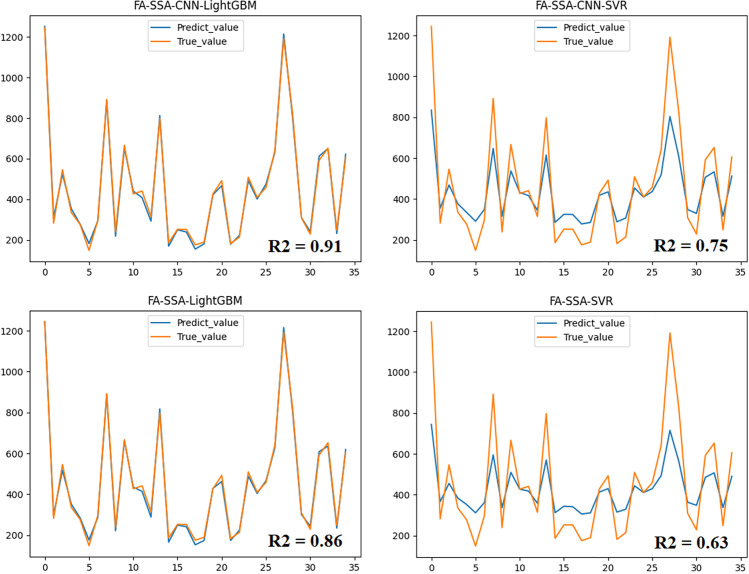
Prediction results of different models.

**Fig 5:**
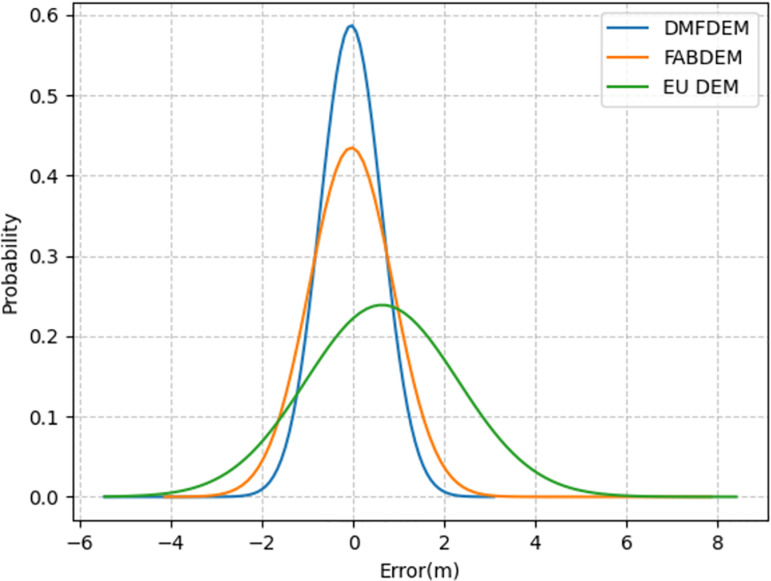
Histogram comparing DMFDEM, FABDEM, EU DEM against the validation data.

**Fig 6:**
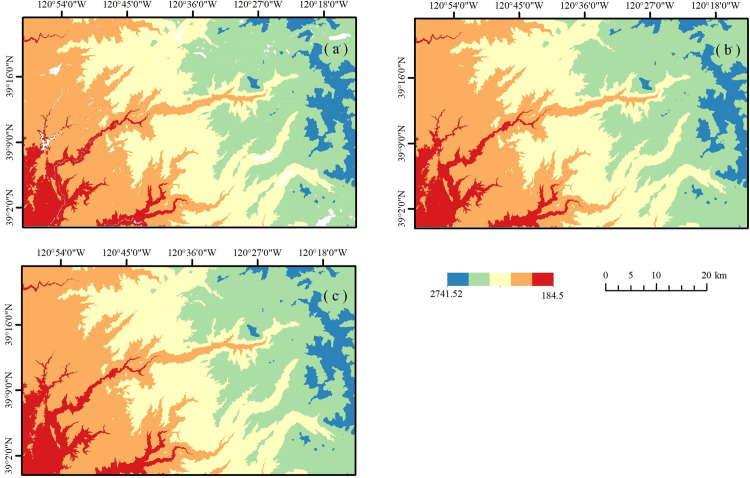
DEM in California (a) DMFDEM; (b) LiDAR DEM; (c) FABDEM.

**Fig 7:**
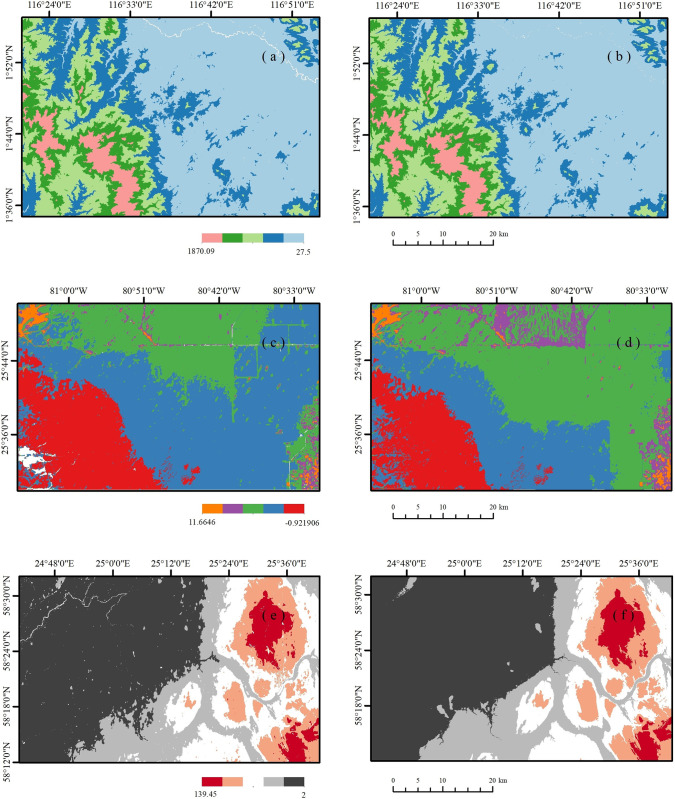
(a) DMFDEM in Kalimantan; (b) FABDEM in Kalimantan; (c) DMFDEM in Florida; (d) FABDEM in Florida; (e) DMFDEM in Pärnumaa; (f) FABDEM in Pärnumaa.

**Fig 8:**
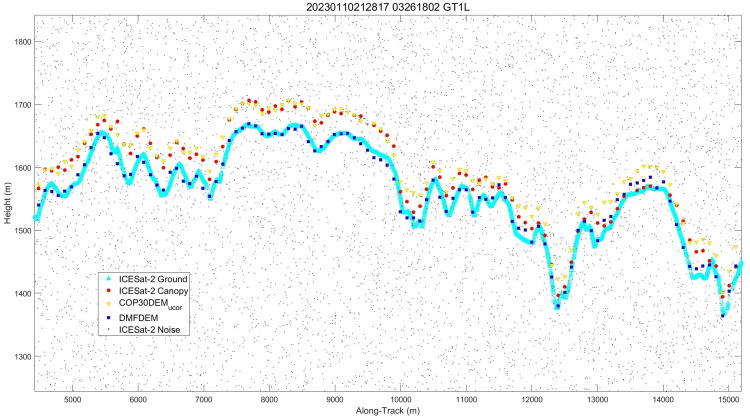
Transect in parts of California.
