# Supplementary material for: LightGBM hybrid model based DEM correction for forested areas
Source: PLoS One. 2024 Oct 7;19(10):e0309025. doi: 10.1371/journal.pone.0309025 (PMC11458030; doi:10.1371/journal.pone.0309025)
Supplement: S1 Appendix — (DOCX) [file pone.0309025.s001.docx]

**Appendix I. Data sources**

ICESat-2 data were downloaded from the NASA National Snow and Ice Data Center (NSIDC) : https://nsidc.org/data/icesat-2.

COP30DEM data were downloaded from the ESA Planetary Data Access (PANDA) : https://panda.copernicus.eu/panda.

Lansat-9 data were downloaded from the United States Geological Survey (USGS) : https://glovis.usgs.gov/app.

Canopy Nico data were downloaded from the ETH Library : https://www.research-collection.ethz.ch/handle/20.500.11850/609802.

Treecover2010 data were downloaded from the Global Land Analysis & Discovery (GLAD) : https://glad.umd.edu/dataset/global-2010-tree-cover-30-m.

Sentinel-2 Land Use/Land Cover data were downloaded from the Environmental Systems Research Institute (ESRI) : https://esri.maps.arcgis.com/apps/instant/media/index.html?appid=fc92d38533d440078f17678ebc20e8e2.

Airborne LiDAR data for the Kalimantan region of Indonesia were downloaded from the Oak Ridge National Laboratory Distributed Active Archive Center (ORNL DAAC) : https://daac.ornl.gov/cgi-bin/dsviewer.pl?ds_id=1540.

Airborne LiDAR data for the Florida and California region of the United States were downloaded from the United States Geological Survey (USGS) : https://apps.nationalmap.gov/downloader/#/.

Airborne LiDAR data for the Pärnumaa region of Estonia were downloaded from the Republic of Estonia Land Board (Geoportal) : https://geoportaal.maaamet.ee/eng/Maps-and-Data/Elevation-data/Download-Elevation-Data-p664.html.
