# Supplementary material for: LightGBM hybrid model based DEM correction for forested areas
Source: PLoS One. 2024 Oct 7;19(10):e0309025. doi: 10.1371/journal.pone.0309025 (PMC11458030; doi:10.1371/journal.pone.0309025)
Supplement: S1 Table — Table 1 lists the mean error (ME), mean absolute error (MAE), and root mean square error (RMSE) of different digital elevation models (DEMs) validated using ICESat-2 data from January to March 2023. (DOCX) [file pone.0309025.s002.docx]

**S1 Table. DEM error verified by ICESat-2.** Table 1 lists the mean error (ME), mean absolute error (MAE), and root mean square error (RMSE) of different digital elevation models (DEMs) validated using ICESat-2 data from January to March 2023.

| Region | Error Metric | DMFDEM | FABDEM | COP30DEM | GEDI |
| --- | --- | --- | --- | --- | --- |
| Kalimantan | ME | **0.42** | -0.59 | 26.07 | -0.91 |
|  | MAE | **1.61** | 1.64 | 26.07 | 2.80 |
|  | RMSE | **1.72** | 1.89 | 26.96 | 3.00 |
| Florida | ME | **0.01** | -0.93 | 5.21 | -1.02 |
|  | MAE | **0.48** | 1.20 | 5.70 | 1.04 |
|  | RMSE | **0.62** | 1.67 | 6.07 | 2.04 |
| California | ME | **-0.26** | 0.52 | 8.64 | 0.82 |
|  | MAE | **1.42** | 1.46 | 9.00 | 1.61 |
|  | RMSE | 1.86 | 1.68 | 10.59 | 3.12 |
| Pärnumaa | ME | **-0.11** | -0.75 | 9.48 | -0.27 * |
|  | MAE | **1.41** | 1.73 | 9.65 | 1.72 * |
|  | RMSE | **1.64** | 1.93 | 10.82 | 2.01 * |
