# Supplementary material for: LightGBM hybrid model based DEM correction for forested areas
Source: PLoS One. 2024 Oct 7;19(10):e0309025. doi: 10.1371/journal.pone.0309025 (PMC11458030; doi:10.1371/journal.pone.0309025)
Supplement: S2 Table — Table 2 shows the datasets used for model training, where ICESat-2 data are considered true values and the rest of the data are training variables. (DOCX) [file pone.0309025.s003.docx]

**S2 Table. Training data and true values.** Table 2 shows the datasets used for model training, where ICESat-2 data are considered true values ​​and the rest of the data are training variables.

| training variables | cop30dem1 | cop30dem2 | cop30dem3 | cop30dem4 |
| --- | --- | --- | --- | --- |
|  | cop30dem5 | cop30dem6 | cop30dem7 | cop30dem8 |
|  | cop30dem9 | L9_B1 | L9_B2 | L9_B3 |
|  | L9_B4 | L9_B5 | L9_B6 | L9_B7 |
|  | L9_B8 | L9_B9 | L9_B10 | L9_B11 |
|  | canopy_nico | veg_cover | land_use | slope |
|  | surface_cutting | relief_amplitude | terrain_roughness | gauss_1 |
|  | gauss_2 | gauss_3 | sobel |  |
| true values | **icesat2** | | | |
